# Targeted Gene Knock-Out of *Fel d1* in Fetal Fibroblasts Using CRISPR–Cas9: Implications for Cat Allergies

**DOI:** 10.3390/ani15070927

**Published:** 2025-03-24

**Authors:** Ling Li, Muhammad Farhab, Paing Oo Kyaw, Xiao-Xiao Xia, He-Qing Cai, Ting Zhang, Ming-Xing Cao, Jin-Gui Li, Yu-Guo Yuan

**Affiliations:** 1 Key Laboratory of Animal Genetic Engineering, College of Veterinary Medicine, Yangzhou University, Yangzhou 225000, China; ll13665200796@163.com (L.L.); farhab.dvm@gmail.com (M.F.); pookyzu2023@163.com (P.O.K.); 18266802391@163.com (X.-X.X.); 16651436946@163.com (H.-Q.C.); 2 Jiangsu Co-Innovation Center of Prevention and Control of Important Animal Infectious Diseases and Zoonoses, Yangzhou University, Yangzhou 225000, China; 3 Laboratory Animal Center, Zhejiang University, Hangzhou 310027, China; 4 Animal Husbandry and Veterinary College, Jiangsu Vocational College of Agriculture and Forestry, Jurong 212400, China; 5 College of Veterinary Medicine, Shanxi Agricultural University, Jinzhong 030801, China; caomingxing@stu.swun.edu.cn

**Keywords:** cat, CRISPR–Cas9, *Fel d1*, gene knock-out, polymorphism

## Abstract

*Fel d1* is the most important allergen secreted by cats, which can trigger asthma in sensitive individuals. Our objective was to knock-out the *Fel d1* gene in the fetal fibroblasts of cats through CRISPR–Cas9 technology with two sgRNAs and to determine the impact of such mutations on the antigenicity of the *Fel d1* protein. DNA samples from 38 domestic cats were collected and amplified by PCR to obtain the complete sequence of the *Fel d1* gene. Throughout evolution, *Fel d1* polypeptide chain 1(*CH1*) has proven to be much more conserved than *Fel d1* polypeptide chain 2(*CH2*); therefore, we targeted *CH2* and designed two single-guide RNAs (*CH2*-sgRNA-1 and *CH2*-sgRNA-2) for this region. Using these constructed sgRNAs, we performed gene knock-out in fetal fibroblasts, resulting in two mutations within the target gene. Following this, DNA was extracted and the target site product was cloned using TA cloning via PCR, and a single colony from this process was sequenced to analyze the physicochemical properties, antigenic sites, and three-dimensional structure of the mutated protein. The results revealed that there were 12 and 51 polymorphic loci (single-nucleotide polymorphisms, or SNPs) found in the *CH1* and *CH2* sequences, respectively, with most loci located in the GC-rich intron 2, while others were found in exon 2, intron 3, and exon 3. These SNPs guided sgRNA design by identifying conserved regions in the *CH2* gene. The gene editing efficiency for the *CH2* region, with this dual CRISPR system, was 40%, with 35% attributed to Type 1 mutation and 5% to Type 2 mutation. In conclusion, *CH1* is significantly more conserved than *CH2*, and the antigenicity of the *Fel d1 CH2* gene in domestic cats can be effectively reduced through CRISPR–Cas9 gene editing.

## Simple Summary

*Fel d1* is the primary allergen released by cats, which is linked to asthma in sensitive individuals. This study aimed to knock-out the *Fel d1* gene using CRISPR technology in cat fetal fibroblasts. Targeting the conserved *CH2* region, two single-guide RNAs were designed, leading to mutations in the gene. This study identified numerous polymorphic loci and achieved a gene editing efficiency of 40%. The findings suggest that the antigenicity of the *Fel d1 CH2* gene can be effectively reduced through CRISPR–Cas9 editing.

## 1. Introduction

Allergenic proteins such as *Fel d1-8* secreted by cats’ sebum, anal, and salivary glands can cause allergic diseases in humans. Among them, the main allergen, *Fel d1* (also called Major allergen I), is a secreted globulin with a size of about 35–40 kDa [1]. It consists of two heterodimers, each with a fraction of 18–20 kDa polypeptide chains named Major allergen I polypeptide chain 1 (*CH1*) and Major allergen I polypeptide chain 2 (*CH2*), which contain allergen sites. The specific biological function is still unclear [2]. As an important source of indoor inhaled allergens, the incidence of human allergic diseases (rhinitis and asthma) caused by *Fel d1* is second only to dust mites in patients with allergic respiratory diseases. The incidence rate in the general population can reach 10–30%, and the respiratory symptoms caused include common rhinitis and severe asthma, which can be life-threatening [3]. *Fel d1* induces IgE reactions in 90–95% of cat allergy patients, and reducing the production of cat allergens is desirable for people with cat *Fel d1* allergies [4,5]. Traditional methods such as using immunotherapy or feeding food containing Igγ antibodies to antagonize *Fel d1* protein in cats to reduce cat allergen production have failed to achieve the desired effect [6,7].

*CH1* and *CH2* are the genes that encode polypeptide chains of *Fel d1* [1]. Recent studies have shown that there are single-base mutations on the *Fel d1* exon of Siberian cats with hypoallergenic properties, and these mutations may be responsible for their hypoallergenicity [8]. At present, the polymorphisms of the *Fel d1* gene in various breeds of cats in China are unclear, and there are no relevant research reports so far. Understanding the specific mutation sequence and polymorphism of the exon of the domestic cat *Fel d1* gene is of great significance for the prevention and control of cat allergies in China and also provides a reference for domestic cat *Fel d1* gene editing.

Transgenic cats expressing GFP protein and other genes have been obtained using traditional transgenic and nuclear transfer technologies [9]. In recent years, CRISPR–Cas9 gene editing technology has been used to achieve efficient gene editing and single-base editing in a variety of animals, such as mice, rats, and livestock [10,11], which is a breakthrough in cat geneediting technology. Using geneediting technology to knock-out the two dimers of *Fel d1* can completely eliminate the production of allergenic proteins, which may be the most effective way to prevent cats from producing allergens at the source [12]. Geneediting technology is used to mutate the *CH1* and *CH2* genes to change the *Fel d1* structure, which may reduce the production of cat allergens, thereby obtaining low-allergen cats. At the same time, the *Fel d1* mutant cat model was used to study the function of the *Fel d1* gene, laying the foundation for research on the pathogenesis of human allergic diseases [13]. Many scientists have explored various methods to eradicate or limit the amount of *Fel d1* in cats, including conjugate vaccine containing recombinant *Fel d18* or egg powder loaded with anti-*Fel d1* antibodies (Immunoglobulin Y) [14]. In 2022, Brackett et al. confirmed that the CRISPR–Cas9 system could eradicate *Fel d1* in feline cells with an invitro transfection study [13]. Lee et al. (2024) developed *Fel d1 CH2* genome-edited cats using the CRISPR–Cas9 system, demonstrating significantly reduced *Fel d1* levels in the *CH2* homozygous edited cat compared to wild-type cats, and achieved successful cloning of the *CH2* homozygous genome-edited cat [15]. Although microinjection into the pronucleus is generally preferred for CRISPR–Cas9 gene editing due to its direct access to the target DNA, enhancing efficiency and reducing off-target effects [16], the cat embryos have the presence of dark lipids in the cytoplasm that made it extremely difficult to microinject the Cas9 and sgRNA mixture directly into the pronucleus [16]. As a result, Lee et al. (2024) opted to microinject the mixture into the cytoplasm instead. Our study aimed to knock-out the *Fel d1* gene in cat fetal fibroblasts using CRISPR–Cas9 with two sgRNAs targeting the *CH2* region, thereby minimizing off-target effects and assessing the impact on *Fel d1* antigenicity.

## 2. Materials and Methods

Nucleotide diversity analysis of *Fel d1* gene was performed by collecting blood samples from 38 domestic pet cats of different breeds (including 8 orange cats, 8 civet cats, 5 calico cats, 2 Linqing lion cats, 2 ragdoll cats, 2 exotic short-haired cats, 4 British shorthair cats, 3 American shorthair cats, 2 Siamese cats, 1 Scottish Fold cat, and 1 Bengal cat) from the animal hospital of the school of veterinary medicine, Yangzhou University, Yangzhou, Jiangsu, China.

### 2.1. Analysis of Nucleotide Diversity of the Fel d1 Gene in Cats

**Figure 1:**
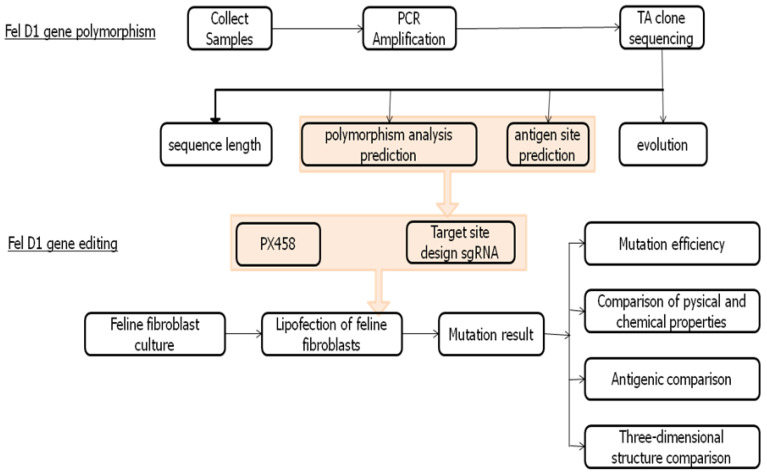
Experiment design. DNA samples from 38 domestic cats were collected and amplified by PCR to obtain the complete sequence of the *Fel d1* gene. We targeted *CH2* and designed two single-guide RNAs for this region, and incorporated these sgRNAs into the PX458 vector, which was used to perform gene knock-out in fetal fibroblasts. It resulted in two mutations within the target gene. Following this, DNA was extracted and the target site product was cloned using TA cloning via PCR, and a single colony from this process was sequenced to analyze the mutation efficiency, physicochemical properties, antigenic sites, and three-dimensional structure of the mutated protein.

DNA was extracted from cat blood to amplify the *CH1* and *CH2* coding sequences of the *Fel d1* gene. During the PCR amplification process, it was found that the conventional PCR method was unable to obtain the target fragment products for *CH1* and *CH2*, or it resulted in products with missing segments, making sequencing and analysis impossible. Subsequently, the *CH1* and *CH2* fragments were successfully amplified by utilizing the Touchdown PCR method and adding 5% DMSO to the PCR system [17] (Table 1 and Table 2). Briefly, 2 × Taq was added at 15 µL for a 1× concentration. An amount of 1.5 µL of All *CH1*F/All *CH2*F and All *CH1*R/All *CH2*R primers were added. DMSO was included at 1.5 µL, resulting in a 5% concentration. An amount of 1 µL Template DNA (200 ng/µL) was added with ddH_2_O to reach 30 µL (Figure 1, Table 1 and Table 2).

Using the primer design software Oligo 7 and Primer 5.0, primers were designed upstream and downstream of the target gene fragments based on the full sequences of *CH1* and *CH2* downloaded from the National Center for Biotechnology Information (NCBI) GenBank database (*CH1*gene ID: 677877, CDS length: 267 nucleotides; *CH2*gene ID: 677879, and CDS length: 324 nucleotides). The primers were named All*CH1* and All*CH2* (Table 1). Following that, the samples were added and mixed according to the PCR reaction system outlined in Table 2. Then, the samples were placed in the PCR machine. The reaction program was set to perform the Touchdown PCR protocol (Table 2 and Table 3). After amplification, 5 µL of the reaction product was loaded into a 1% agarose gel containing a nucleic acid dye for electrophoresis. After electrophoresis, the gel was photographed in the BioRad Gel Doc system. If there were DNA bands corresponding to the size of the target, the gel was cut, and the DNA was recovered to perform TA cloning, or stored at −20 °C for future use.

### 2.2. TA Cloning and Sequencing

We employed TA cloning to isolate and sequence individual mutant alleles generated by CRISPR–Cas9 editing, yielding definitive and unambiguous data about the nature of the mutations transferred, which is important for delineating the range of mutations and assessing the efficiency of gene editing, especially when dealing with heterogeneous populations of edited sequences.

#### 2.2.1. Ligation

The DNA recovered from the (electrophoresis) gel was prepared with the 5 min TA/Blunt-Zero Cloning Kit (Vazyme, Nanjing, China) as per the manufacturers’ recommendations (Table 4). All liquid was collected by centrifugation, and the centrifuge tubes were placed in a room (above 20 °C) or in the PCR machine at 25 °C for 5 min to maintain temperature.

#### 2.2.2. Transformation

The ligated plasmid product was added to the competent cells and gently mixed. The mixture was kept on ice for 30 min, subjected to a heat shock at 42 °C for 45 s, and then returned to ice for 2 min. Next, 500 µL of antibiotic-free LB bacterial broth was added to the centrifuge tube, and the culture was incubated at 37 °C with shaking at 200 rpm for 1 h. After incubation, 100 µL of the cultured bacteria was plated onto an LB solid agar plate containing ampicillin, and the plate was spread evenly with a sterile spreader before being placed inverted in a 37 °C incubator for 1 h. It was then incubated upright for 12–15 h. The single colonies that appeared on the plate were transferred into an LB liquid culture tube containing ampicillin. This tube was placed in an incubator shaker at 37 °C and 200 rpm for 12–16 h.

#### 2.2.3. Sequencing

The primer design software Oligo 7 and Primer 5.0 were used to design two pairs of primers, specifically at their second exons (Table 1), as the antigenic site of *Fel d1* is located near or after exon 2; targeting exon 2 is therefore more likely to impair the protein’s structure and function. Primers *CH1*-E2-1 and *CH2*-E2-1 were synthesized. PCR was performed using a 20 µL system (Table 5 and Table 6), followed by gel electrophoresis and sequencing of positive colonies.

The results of the sequencing obtained for the *CH1* and *CH2* genes of the cat *Fel d1* protein were analyzed using SnapGene 8.0 software. Comparison analysis of the base sequences obtained from sequencing was conducted using MEGA11.

SNPs have little influence on the generation of allergen-free cats since sgRNA could be easily designed to target any SNP in the corresponding cat breed to knock-out *Fel d1*. However, it is generally more practical to design sgRNAs targeting conserved regions of the *Fel d1* gene across multiple cat breeds rather than designing breed-specific sgRNAs to account for SNPs. The number of bases, SNPs, and insertions and deletions (indels) for each sequence were calculated using DNASP5.0 software. The intronic portions of the sequences were then excluded, and the nucleotide diversity (Pi), SNPs, and average nucleotide difference (k) of the gene coding region were calculated.

### 2.3. Fetal Fibroblast Culture

To perform and evaluate the geneediting efficiency of targeting vectors, orange cat fetal fibroblasts were cultured in our laboratory, a method adapted from Seluanov et al. (2010) [18]. Briefly, we obtained the aborted fetus sample, which was not more than 4 h old, and placed it on the ice at 4 °C. The fetus was then submerged in 70% ethanol for 10 min, followed by washing with normal saline three times. The fur around the incision area was shaved and 70% ethanol was applied to the desired incision site of the connective tissue, allowing the ethanol to evaporate. Then, connective tissue was excised and approximately 1 cm^2^ samples were taken, and transferred into a 10 cm tissue culture dish. The samples were cut into pieces of approximately 1 mm and then trypsinized with 0.05% trypsin (Sigma-Aldrich, St. Louis, MO, USA) for 10 min at 37 °C, 5% CO_2_. We pipetted the trypsin to break up clumps, centrifuged the solution, and resuspended the pellet in fresh media to stop the digestion. This process was repeated to ensure no residual enzymes remained. Finally, we plated the resuspended cells in RPMI-1640 media supplemented with 10% fetal bovine serum (FBS), penicillin (0.1 mg/mL), and streptomycin (0.1 mg/mL) in a tissue culture dish and incubated it at 37 °C, 5% CO_2_. After 7 days, we changed the media and transferred tissue fragments to new plates if necessary. By day 14, all viable fibroblasts had exited the tissue fragments. We then harvested the cells, plated them at 5 × 10^5^ cells per plate in RPMI-1640 with 10% FBS, penicillin (0.1 mg/mL), and streptomycin (0.1 mg/mL), and froze an aliquot for future use. We continued culturing the cells by splitting them at 80–90% confluence. Our representative results showed that normal fibroblasts appeared as large cells with prominent protrusions, growing in a monolayer. A healthy culture contained 1–10% cells in the mitotic (M) stage, recognizable as rounded-up cells elevated over the plate surface. Typically, a 10 cm dish seeded with 5 × 10^6^ cells became confluent in 4 days.

### 2.4. Transfection of Fetal Fibroblasts for Gene Knock-Out

At 100% confluence, these orange cat fetal fibroblasts were transfected with vectors (*CH2*-sgRNA-1 and *CH2*-sgRNA-2) using Lipofectamine 3000 as follows: To prepare the transfection mixture, two sterile 1.5 mL centrifuge tubes were placed. An amount of 125 µL of Opti-MEM medium was added to each tube. An amount of 2.5 µg of plasmid DNA (1.25 µg of PX458-*CH2*-sgRNA-1 and 1.25 µg of PX458-*CH2*-sgRNA-2) was added to one tube, and the mixture was gently pipetted to combine. An amount of 5 µL of liposomal transfection reagent (Hieff, Yeasen, Shanghai, China) was added to the other tube, and the mixture was gently pipetted to combine. The 125 µL of Opti-MEM medium containing the plasmid DNA was gently added to the centrifuge tube containing the transfection reagent. The mixture was pipetted to combine and then incubated at room temperature for 15 min. The incubated plasmid DNA and liposome complex were added to the cell culture wells of the six-well plate. After 6–8 h, the culture medium was replaced with fresh culture medium. The transfection status was observed under a fluorescence microscope 24–36 h later. After 48 h, the GFP status of the cells was observed under a fluorescence microscope.

### 2.5. Physicochemical Properties and Structural Analysis of Mutant Fel d1 Protein

To evaluate the physicochemical properties and structural analysis of mutant *Fel d1* protein, we carried out RNA simulation transcription and protein translation of the sequence of the mutated potential antigenic site and used DNASTAR lasergene 11.1 software to analyze the physical and chemical properties and antigenicity of its coding sequence, including hydrophilicity, surface accessibility, antigenicity, plasticity, and dioxin level structure dependencies. We used the online website www.expasy.org/resources/swiss-model (accessed on 10 September 2024) to perform homology modeling and compared the structural changes in the wild sequence after base deletion and the edited coding sequence.

## 3. Results

### 3.1. Amplification Results of Cat Genes

**Figure 2:**
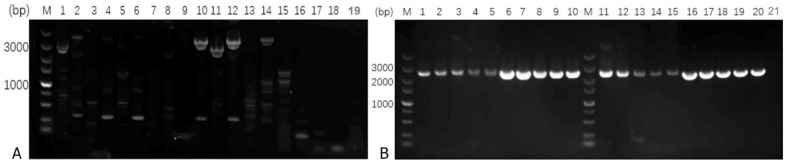
Electropherogram of *CH1* and *CH2* genes from different cat blood DNA samples. (**A**) Before PCR optimization. Lanes 01–10: *CH1* gene, only lane 1 showsa band corresponding to the target size; lanes 11–19: *CH2* gene, only lane 11 shows a band corresponding to the target size. (**B**) After PCR optimization. All lanes show a band corresponding to the target size. Lanes 01–10: *CH1* gene; lanes 11–20: *CH2* gene; lane 21: negative control; bp: base pairs;and M: DL5000 DNA Marker.

After amplifying the entire sequences of the *CH1* and *CH2* fragments from the extracted cat blood DNA, 5 µL of the product was subjected to 1% agarose gel electrophoresis. The target band was excised and subjected to TA cloning, and single clones were picked for sequencing. The sequencing results further confirmed the successful amplification of the *CH1* and *CH2* fragments. Touchdown PCR amplification results of *CH1* and *CH2* genes using optimized conditions are presented in Figure 2.

### 3.2. Sequence Length of Each Region of the Fel d1 Gene

The sequences that were obtained from the Chinese domestic cats were compared against the reference (wild-type) sequences [15] by utilizing SnapGene software (www.snapgene.com) in order to search for any variation. All successfully cloned sequences have three exons and two introns, and no mutation was observed within the exon1, intron1, and exon2 of the *CH1* and *CH2* genes. Exon 2 of the *CH1* gene and intron 2 and exon 3 of the *CH2* gene have indels, as shown in Table 7. However, these indels were not present in all samples but did present in some individuals and thus constituted a variable presence across the samples.

### 3.3. Analysis of Nucleotide Diversity of the Fel d1 Gene

By comparing and analyzing the data of nucleotide diversity distribution of the *CH1* chain of the *Fel d1* gene, it was found that there was no nucleotide diversity in exon 1, intron 1, and exon 3 within the *CH1* gene, while exon 2 and exon 3 were mutated. In intron 2 of the *CH2* gene, only exon 1 with a length of 68 bp remained conserved, while all other sites were mutated, with the most obvious mutation observed in exon 2. These results showed that the mutation within the *CH2* gene was significantly higher than that of *CH1* (Table 7 and Table 8).

### 3.4. Analysis of Fel d1 Gene Polymorphism Sites

The Sanger sequencing was analyzed through DNASP 5.0 software, and the parameters under consideration were the number of invariant sites, SNPs, parsimonious information sites, and indels in each region (exons and introns) of the *CH1* and *CH2* peptides. As shown in Table 7, the region with the most SNPs detected in *CH1* and *CH2* is located on intron 2, with 7 and 23, respectively. There are 2 SNP sites in *CH1* exon 2, and the *CH2* sequence has a total of 31 SNP sites in other regions. In addition, it was also found that the *CH1* sequence in the cat population has 1 InDel site, located on intron 2; the *CH2* sequence in the population has 15 InDel sites, located on intron 2 and exon 3. The results further prove that *CH2* sequences are more prone to mutations and more diverse than *CH1*.

### 3.5. Polymorphism Analysis of Fel d1 Coding Sequence

By calculating the nucleic acid diversity (Pi) and the average number of nucleotide differences (K), SNPs, and indels, we found that the *CH2* gene diversity is much higher in the coding region on *CH1*, as shown in Table 8.

### 3.6. Phylogenetic Analysis of the Fel d1 Gene

**Figure 3:**
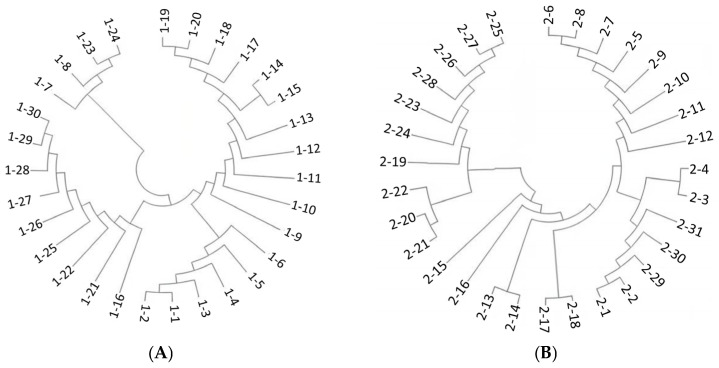
Evolutionary map of the (**A**) *CH1* gene and (**B**) *CH2* gene in different domestic cats.

A total of 30 coding sequences of the *CH1* gene and 31 coding sequences of the *CH2* gene were amplified. After sorting and comparing each coding sequence through MEGA11 software, phylogenetic trees of *CH1* and *CH2* genes were drawn and are presented in Figure 3A,B. It can be found that *CH1* genes 1–16 and 2–17 are located in the center of the phylogenetic tree. After comparison, it is found that these two sequences have no differences compared with the reference RNA sequence on NCBI (*CH1*: NM_001048153.1; *CH2*: NM_001048154.1). In the case of deletions or mutations, each branch has more or less mutations or deletions of individual bases, which reveals the evolution of the gene.

### 3.7. Fel d1 Gene Editing Through the CRISPR–Cas9 System

**Figure 4:**
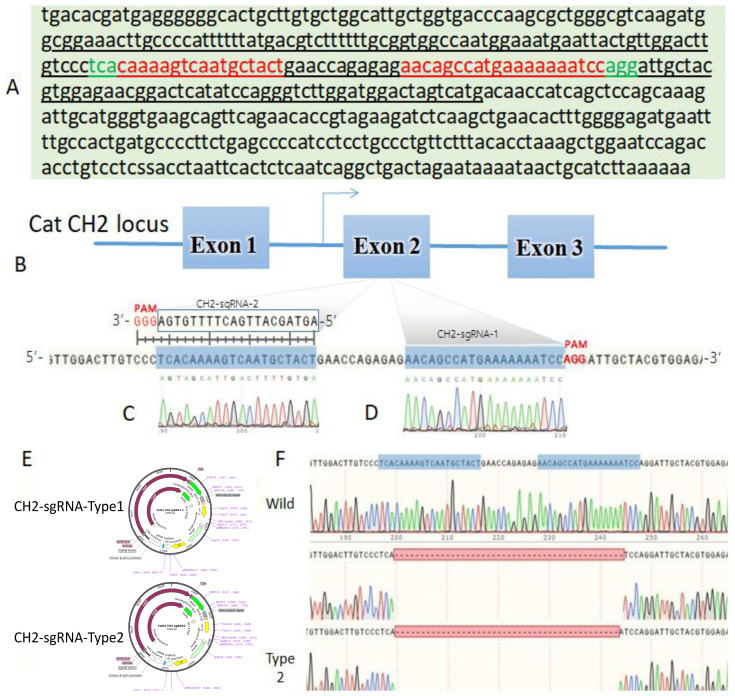
Schematic diagram of *CH2* genes depicting the amplified and sequenced gene regions. (**A**) The *Fel d1* sequence was retrieved from https://www.ncbi.nlm.nih.gov/protein/NP_001041619.1. (accessed on 10 September 2024) Single-underlined nucleotide sequences represent exon 2. The black underline indicates exon 2 of *CH2*. The red underline indicates target sgRNA candidates within exon 2, the green nucleotides indicate PAM sequences within exon 2, and the red nucleotides indicate sgRNA candidates. The complement to the left-side green nucleotides is *CH2*-sgRNA-2 (PAM: tca) and the complement to the right-side green nucleotides is *CH2*-sgRNA-2 (PAM: agg). (**B**) *CH2* gene targeting site. (**C**) Sanger sequencing peaks of *CH2*-sgRNA-1. (**D**) Sanger sequencing peaks of *CH2*-sgRNA-2. (**E**) Map of the constructed PX458-*CH2*-sgRNA plasmids. (**F**) Mutations within the target sequences compared to the wild type.

The successfully constructed *CH2* sgRNAs (Figure 4) were also successfully ligated with the T4 DNA ligase and BbsI of the pX458 plasmid. The constructed plasmids were named *CH2*-sgRNA-Type1 and *CH2*-sgRNA-Type2 and are presented in Figure 4E.

For *CH2*-sgRNA-1 (Type 1), *CH2*-sgRNA-2 (Type 2), and *CH2*-E2, the primers designed are presented in Table 1. The PCR reaction system specifications were 200 ng DNA template, 0.5 pmol forward primer (10 µmol/L), 0.5 pmol reverse primer (10 µmol/L), 15 μL 1×Taq, and up to 30 μL ddH_2_O (Table 1).

The Touchdown PCR reaction procedure was divided into three phases. In phase 1, we performed the initial denaturation at 95 °C for 5 min, denaturation at 95 °C for 1 min, annealing at 65 °C for 45 s, and elongation at 72 °C for 3 min. Denaturation, annealing, and elongation were repeated twenty times (cycles), and each time the annealing temperature was decreased by 0.5 °C. In phase 2, we performed the initial denaturation at 95 °C for 1 min, annealing at 55 °C for 45 s, and elongation at 72 °C for 3 min. Phase 2 was repeated twenty times (or cycles). In phase 3, we performed the elongation at 72 °C for 10 min and then stored the PCR product at 4 °C until use (Table 8).

We then sequenced the PCR products through DNA extracted from the 1% agarose gel for target site sequencing. A set of peaks appeared near the target sequence, indicating that there was a base fragment mutation or deletion at this site. The PCR products with set peaks were purified and TA-cloned for further sequencing verification. Obtained sequences were analyzed through Molecular Evolutionary Genetics Analysis (MEGA) software version 11 [19]. The redundant sequences at both ends of the coding region were removed, and only the base sequences of the coding region and introns were retained. We then calculated the nucleotide diversity (Pi), SNPs, and average nucleotide differences (k) of the coding region of the gene as presented in Table 7 and Table 8.

Our objective was to identify, alter, or eliminate the *Fel d1* antigenic epitope in order to potentially avoid allergic reactions by blocking the *Fel d1* protein from binding to human antigen receptors. Thus, we built sgRNA that specifically targeted the antigenic epitope’s location (Figure 4). A Protein Data Bank (PDB) was used to access the structural data of *Fel d1*, and its antigenic epitopes, secondary structure, surface flexibility, hydrophilicity, and surface accessibility were checked through DNASTAR lasergene 11.1 software. We used the sgRNA online tool (https://crispor.gi.ucsc.edu/) (accessed on 10 September 2024) to design the target site on the cat *CH2* exon antigen site. T4 DNA ligase and BbsI were used to ligate the sgRNA with the pX458 plasmid. We then transformed the constructed plasmid into DH5α competent cells and sequenced them for confirmation of plasmid construction (Figure 3A,B). Successfully constructed plasmids were named *CH2*-sgRNA-1 and *CH2*-sgRNA-2, but we used only *CH2*-sgRNA-2 for experimentation, as presented in Figure 4E.

**Figure 5:**
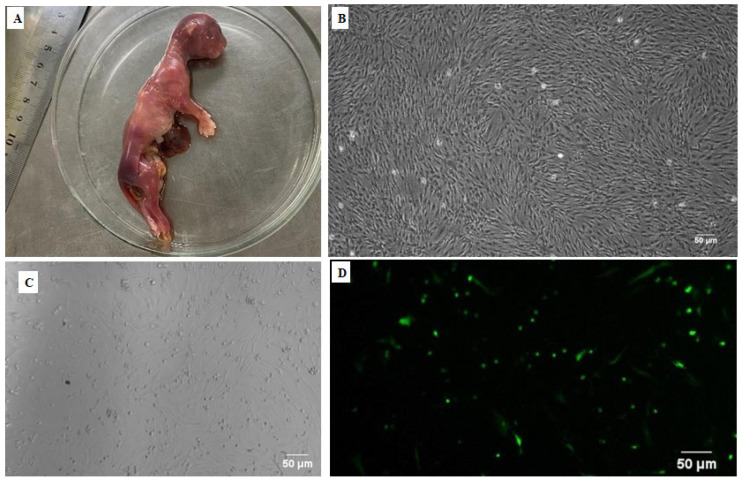
(**A**) Aborted fetus of a domestic cat; (**B**) domestic cat fetal fibroblasts cultured from an aborted fetus. Fluorescence was measured 36 h after transfection from (**C**) bright-field microscopy and (**D**) fluorescent microscopy.

Fetal fibroblasts were successfully cultured from orange cats in our laboratory as presented in Figure 5A,B, and were successfully transfected with constructed vectors (*CH2*-sgRNA-1 and *CH2*-sgRNA-2) using Lipofectamine 3000, as confirmed by the presence of green fluorescent protein (GFP) after 48 h of transfection as presented in Figure 5C,D.

### 3.8. Targeting Vector Mutation Analysis

**Figure 6:**
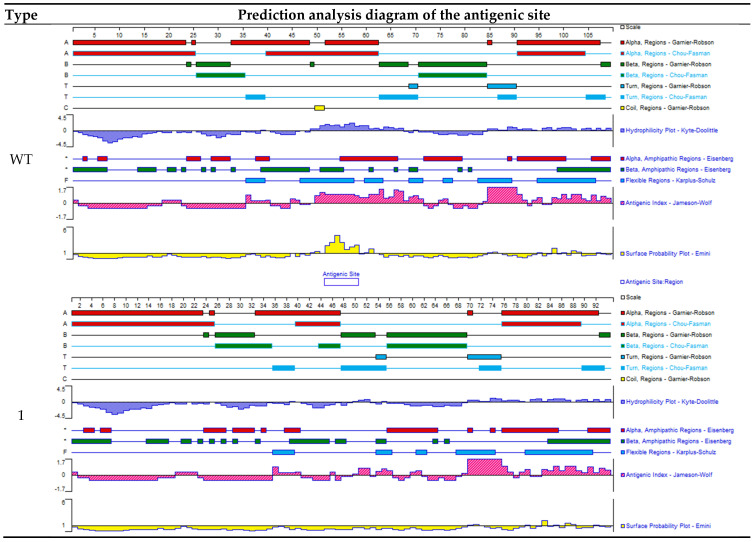

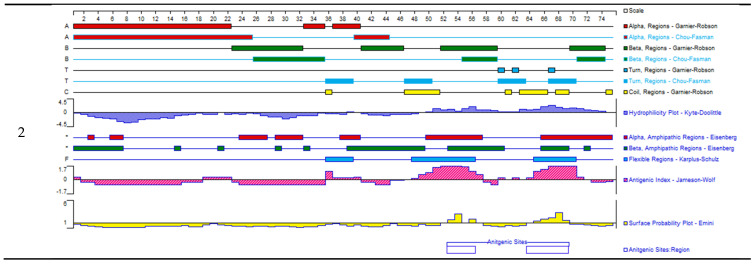
Indels, their frequency (F), prediction analysis diagram of the antigenic sites, and three-dimensional structure of the wild-type and mutant *Fel d1* encoded protein. The surface probability plot (Emini) detects the regions of high surface accessibility, and these regions would be potential surface-exposed regions of the protein. The wild-type (WT) protein has an antigenic site at amino acids 53–58. The Type 1 mutation has no significant antigenic sites. The Type 2 mutation has antigenic sites at amino acids 53–57 and 64–69 (created through DNAStar Protean).

**Figure 7:**
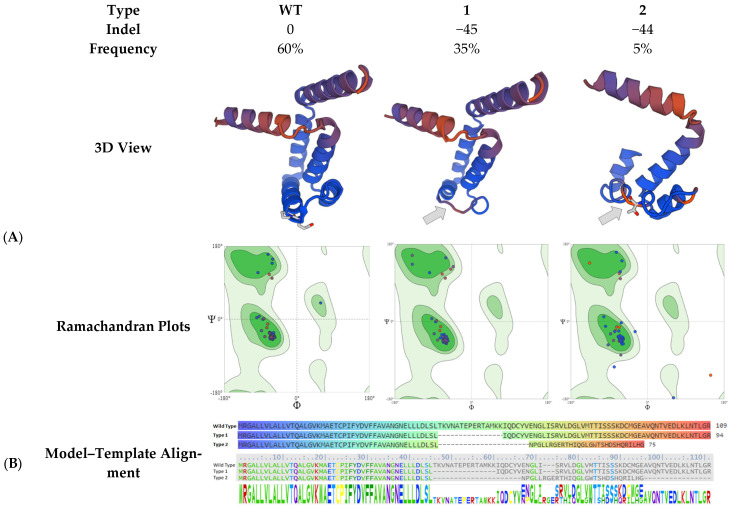
Analysis of the sequencing results of 20 single clones showed that 12 clones had no mutations (frequency = 60%); 7 clones were missing 45 bases near the target site (frequency 35%); and 1 clone was missing 44 bases near the target site (frequency 5%). (**A**) Three-dimensional (3D) structure of the encoded protein. Template: P30440.1. A Major allergen I polypeptide chain 2 AlphaFold DB model of FEL1B_FELCA (gene: *CH2*, organism: *Felis silvestris catus*). In the Ramachandran Plot, wild type and Type 1 have stable structures, but Type 2 represents two amino acids falling in regions of unusual structural features, i.e., one blue dot representsamino acid 61 within the right bottom quartile, and one red dot representsamino acid 57 within the right-handed-alfa-helix quartile (Created through https://swissmodel.expasy.org/interactive/ (accessed on 10 September 2024)). (**B**) Comparison of the amino acid precursor sequence of gene-edited *Fel d1* (*CH2*) (5′ → 3′).

The cells transfected with the plasmid were subjected to Fluorescence-Activated Cell Sorting (FACS) screening to isolate GFP-positive cells, and DNA was extracted from the green fluorescent protein (GFP)-positive cells for target site sequencing. The results are shown in Figure 5D, A set of peaks appeared near the target sequence (Figure 4F, indicating that there is a base fragment mutation or deletion at this site. The PCR products with set peaks were purified and TA-cloned for further sequencing verification. A total of 23 single clones were sent, and 20 sequencing results were successfully obtained. Analysis of the sequencing results showed that 12 clones had no mutations, with a rate of 60%; sevenclones were missing 45 bases near the target site, with a mutation efficiency of 35%; and one clone was missing 44 bases near the target site, with a mutation efficiency of 5% (see Figure 6 and Figure 7). The preliminary results indicate that the two designed sgRNAs significantly knocked out the target sequence.

### 3.9. Physicochemical Properties and Structural Analysis of Mutant Fel d1 Protein

The physical and chemical properties of the encoded proteins of the three sequencing results were analyzed and compared through DNASTAR Protean. The amino acid sequence after mutation is shown in Figure 6 and Figure 7. Compared with the wild-type sequence (12/20 clones, frequency = 60%), Type 1 lacked 45 nucleotide acid–base pairs and was deficient in 15 amino acids (7/20 clones, frequency = 35%). The specific deletion is shown as “-” in the red sequence in the Type 1 and Type 2 sequences (Figure 6). Type2 has 44 nucleic acid–base pair deletions (1/20 clone, frequency = 5%). All the amino acids on the 3’ of the mutation in Type2 were also changed, which resulted in 32 amino acid deletions compared with the wild type.

We performed the prediction analysis of antigenic sites using the Emini surface probability plot to detect regions of high surface accessibility, which are potential surface-exposed regions of the protein. As shown in Figure 6, the wild-type (WT) protein has an antigenic site at amino acids 53–58. After knocking out the potential antigenic site, Type 1 has 45 bases deleted, and the antigenicity of the overall sequence was significantly reduced compared with the wild type (WT). This theoretically proves that the mutated *Fel d1* protein Type 1 has reduced allergenicity. Type 2 lacks 44 nucleotide bases, and the resulting frameshift mutation in the DNA sequence causes premature translation termination, resulting in a reduction of 32 amino acids in the overall peptide chain compared to the wild sequence. At the same time, two possible potential antigenic sites (marked by blue boxes) appeared downstream of the deleted site at amino acids 53–57 and 64–69, but the antigenic site at 64–69th amino acids may not be associated with allergenicity, as the protein structure after the 57th amino acid is disrupted compared to the wild-type sequence (Figure 6).

We compared the amino acid sequence of the gene-edited *Fel d1* (*CH2*) with the wild type. The three-dimensional structural model of wild-type and base-deleted *Fel d1* proteins was composed of α-helices and turning angles. After deleting 45 bases and 15 amino acids as “TKVNATEPERTAMKK”, the overall sequence structure complexity of Type 1 was reduced, and the complexity of the α-helix and β-turn junction shown by the arrow was significantly reduced, confirming the result of reduced antigenicity. Deletions of 44 bases from Type2 have led to reduced overall size compared with the wild type and Type1, along with the position of α-helices being upstreamed. In Type 2, disruption of codons downstream to the sgRNA target site led to frameshifts and a premature stop codon, resulting in gene knock-out (Figure 7B).

We used the Ramachandran Plot to assess the stability of the mutated structures. We found that wild type and Type 1 have stable structures, but Type 2 represents two amino acids falling in regions of unusual structural features, i.e., one blue dot representing amino acid number 61 within the right-handed alpha-helix quartile and one red dot representing amino acid number 57 within the right bottom quartile. Both of these amino acids are downstream of the sgRNA target site, providing evidence of an unstable three-dimensional protein structure (Figure 7A).

Homology modeling was performed on the mutant *Fel d1* protein, and the three-dimensional structure is shown in Figure 7 as a 3D view. Model–template alignment was added to check the amino acid within the 3D structure, as the same color of the model template sequence and 3D protein structure correspond to the same amino acid. Arrows on Type 1 and Type 2 indicate the regions where sgRNAs were designed. The Type 1 3D structure is conserved downstream to the sgRNA site, while it is disrupted in Type 2. The results of indels of the amino acid precursor sequence of the gene-edited *Fel d1* (*CH2*) are presented in Figure 7B.

## 4. Discussion

Cats are the principal producers and disseminators of fatal human respiratory allergens. So far, there is a lack of detailed research on the *Fel d1* gene polymorphism of domestic cats in China. This article collected and amplified the *Fel d1* gene of 38 domestic cats and found that there are mutations and polymorphic sites in both *CH1* and *CH2* genes. Some of these mutations exist in exons, leading to changes in the encoded proteins. There are two polymorphic amino acid sites on the *CH1* coding chain. The 47th amino acid is mutated from lysine to asparagine, and the 78th amino acid is mutated from leucine to valine. There are five polymorphic amino acid sites on the *CH2* coding chain, including the mutation of phenylalanine at position 32 to tyrosine; lysine at position 38 to arginine; and valine at position 45 to leucine acid. Moreover, histidine at position 72 is changed to lysine and glycine at position 75 is mutated to valine. These mutation results differ from the *CH1* and *CH2* polymorphism results observed in the four Siberian cats examined in a study. Perhaps the number of samples and regional differences led to the differences in the results [8]. Studies have shown that natural variation in *CH1* and *CH2* genes has nothing to do with the expression level of the *Fel d1* protein [20]. Additionally, other studies have also reported that the expression levels of protein-related RNA vary greatly between tissues, and the sites with the most abundant RNA expression are salivary glands and sebaceous glands [13].

When analyzing and comparing the obtained sequencing results, the regions carrying multiple SNPs, parsimony informative sites, and indels in *Fel d1* were all located in intron 2. Intron 2 is the site with the highest GC content and repetitive sequences in the entire sequence. Some of the sequences have GC content of 65% and above. It is not only difficult to amplify in vitro, but the final sequencing results also show that indel polymorphisms are also mostly concentrated in this region, which are indicators of their significance in maintaining the structure and stability of the two DNA strands. The specific function of these SNPs and indel polymorphisms needs to be confirmed by further research. A full sequence homology search of the *Fel d1* gene through NCBI found that a specific protein in mice, Androgen Binding Protein (ABP), has 35–60% homology with the *Fel d1* protein sequence of domestic cats. This mice protein is involved in mate selection and information exchange between mice [13]. Further experiments showed that gene knock-out of ABP had no obvious effect on the growth and development of mice, suggesting the safety of cat *Fel d1* protein editing [21]. Analysis of data collected from different cats in this study shows that the polymorphic site of *CH2* in *Fel d1* is significantly higher than that of *CH1*, suggesting that *CH2* may have a less critical biological function compared to *CH1*, and conservative sequences should be selected when performing gene editing. Editing as a target site is beneficial to the stability of experimental results and reducing off-target effects. Therefore, in the subsequent experimental design, selecting the conserved site on *CH2* as the target site can reduce the impact of gene editing on the survival and development of felines.

A single CRISPR system, that is, a sgRNA and Cas9 protein, can cause fragmentation of the blunt ends of DNA in the target sequence, causing random insertion and deletion of bases at the target site [22,23]. Designing two target sites, that is, two sgRNAs and Cas9 protein, can cause the deletion of the target fragment [22]. There are currently few studies on gene editing in cat genomes. In this study, although the potential antigenic site in the *Fel d1* gene was successfully knocked out by simultaneous transfection of two plasmids, with a gene editing efficiency of 40% indicating that the designed sgRNA has strong cleavage activity, further validation through the generation of gene knock-out cats was not performed, as it falls outside the scope of this study. Brackett et al. (2022) designed 10 sgRNAs and used the CRISPR system to edit the *Fel d1* gene of cat kidney epithelial cells. The editing efficiency was between 5 and 55%, which is similar to the results of this experiment [13]. By analyzing potential off-target sites in the entire cat genome, the two sgRNAs in this experiment did not have potential off-target sites.

Brackett et al. (2022) [13] identified conserved coding regions in *CH1* and *CH2* that are suitable for CRISPR editing. Their comparative analyses revealed relatively low sequence identities for *CH1* and *CH2* across species, suggesting that *Fel d1* may be nonessential. The relatively low sequence identities also question the efficiency of sgRNAs in different breeds of cat, raising important questions about the design of sgRNAs for *Fel d1* editing in every breed of cat, which is not only capable of producing *Fel d1*-negative cats but also exhibits no detectable off-target effects that could negatively impact cat health or physiology. Our study takes the field to the next level by reporting population-specific genetic data on *Fel d1* in domestic cats in China, demonstrating CRISPR–Cas9 editing in fetal fibroblasts and structural and functional analysis of *CH2* gene mutations that effectively reduce the antigenicity of *Fel d1*. The findings set the stage for future in vivo studies and the creation of hypoallergenic cats.

In our study, we analyzed the structural and physicochemical properties of the mutated *Fel d1* protein primarily to understand the potential consequences of the observed mutations on protein function and allergenicity. While the majority of CRISPR-induced mutations are indeed frameshifts (Figure 7B, Type 2), we observed a subset of mutations that resulted in in-frame deletions (Figure 7B, Type 1). These are in vitro strong suggestions that the edited *Fel d1* gene is very likely to be non-functional. This assay was included to ascertain whether even partial modifications of the protein could render it less allergenic. However, the final verdict on *Fel d1* function would come from the generation of knock-out cats and functional assays such as its binding with human IgE antibodies or its role in lipid binding or immune modulation, if any.

Recently, Lee et al. (2024) generated *Fel d1 CH2* gene-edited cats using CRISPR–Cas9 [16]. They microinjected the mixture of sgRNA from the firstexon and Cas9 mRNA into the cytoplasm of one-cell stage cat embryos. They faced a significant challenge in their experiments involving cat one-cell stage embryos. The presence of dark lipids in the cytoplasm made it extremely difficult to microinject the Cas9 and sgRNA mixture directly into the pronucleus, a step that is typically preferred for its precision and efficiency in gene editing. As a result, they opted to microinject the mixture into the cytoplasm instead. This approach, while less precise, allowed them to bypass the visual challenges posed by the embryos’ composition and still achieve gene editing, albeit potentially with lower efficiency and higher risk of off-target effects. In contrast, our study focused on targeting the *CH2* region with two sgRNAs, which in challenging contexts like cat one-cell stage embryos may minimize off-target effects. Utilizing ELISA, their results on cat fur and saliva indicated that *CH2* genome-edited founder parent cats and the *CH2* homozygous genome-edited cat showed low levels of *Fel d1*, with the homozygous cat exhibiting exceptionally low levels [16]. They detected no off-target effects. However, their findings also highlight the need for further exploration of the “optimal design” of sgRNAs to ensure consistent and safe gene editing across different cat breeds.

## 5. Conclusions

This study analyzed the polymorphisms and structural characteristics of the *Fel d1* gene and protein of several strains of cats, and successfully mutated the *Fel d1 CH2* gene of domestic cat fetal fibroblasts, knocking out part of the antigenic sites of the *Fel d1* protein. Our dual-sgRNA CRISPR–Cas9 approach is a promising step toward mitigating the allergenic potential of *Fel d1* in household cats. Further optimization, though, including evaluation of additional sgRNAs and consideration of natural genetic variation, will be required to optimize the efficacy and practicality of this approach. This will lay the foundation for the later use of CRISPR–Cas9 to obtain hypoallergenic cat breeds.

## Figures and Tables

**Table 1:** Touchdown PCR Primer information.

Primer Name	Direction	Primer Sequence	Tm (°C)	Destination Fragment Length (bp)
All *CH1*	F	AGACCGGCCTCCTTTTTGGTA	58	2499
	R	GCCACCTGTGCTAATGGTCA	59	
All *CH2*	F	TGTCACCTCCCTTTGCCAT	57	2462
	R	CTCTTAACAGCCCAAGGGT	57	
*CH1*-E2-1	F	CCAGGGCACTTCTGAGCAT	59	443
	R	ATGAGTGCCCCTCTACTTCC	57	
*CH2*-E2-1	F	TCTCAAGTGGCGGGCAAACA	59	427
	R	AGGTTGCGTATTCTATGTGCT	57	

F: forward primer; R: reverse primer; and Tm: melting temperature of primer sequence.

**Table 2:** Touchdown PCR reaction system.

Reagents	Adding Volume (µL)	Final Concentration
2 × Taq	15	1×
All *CH1*F/All *CH2*F (10 µmol/L)	1.5	0.5 μmol/L
All *CH1*R/All *CH2*R (10 µmol/L)	1.5	0.5 μmol/L
DMSO	1.5	5%
Template (200 ng/µL)	1	
ddH_2_O	Upto 30	

**Table 3:** Touchdown PCR reaction procedure.

Steps	Reaction Program	Temperature (°C)	Time	Number of Cycles
1	Pre-denaturation	95	5 min	1
2	Denaturation	95	1 min	20 (each cycle reduces the annealing temperature by 0.5 °C)
	Annealing	65	45 s	
	Extension	72	3 min	
3	Denaturation	95	1 min	20
	Annealing	55	45 s	
	Extension	72	3 min	
4	Extension	72	10 min	1
5	Storage	4	Forever	

**Table 4:** TA cloning system.

Reagents	Volume
5 × TA/Blunt-Zero Cloning Mix	1 µL
Gel recovery DNA	100 ng
ddH_2_O	Upto 5 µL

**Table 5:** Procedure for detecting the PCR reaction system.

Reagents	Adding Volume (µL)	Final Concentration
2 × Taq	10 µL	1×
*CH1*-E2-1F/*CH2*-E2-1F (10 µmol/L)	1 µL	0.5 μmol/L
*CH1*-E2-1R/*CH2*-E2-1R (10 µmol/L)	1 µL	0.5 μmol/L
Bacterial liquid	1 µL	
ddH_2_O	Upto 20 µL	

**Table 6:** Procedure for detecting PCR products.

Reaction Steps	Temperature (°C)	Time	Number of Cycles (Times)
Pre-denaturation	95	5 min	1
Denaturation	95	50 s	
Annealing	55	30 s	35
Extension	72	45 s	
Final extension	72	10 min	1
Storage	4	Forever	

**Table 7:** Analysis of the nucleotide sequence lengths and polymorphic sites in the *Fel d1* gene between template and population sequences.

*Fel d1 CH1*	Parameter	Total Sequence	Exon 1	Intron 1	Exon 2	Intron 2	Exon 3
Nucleotide sequence lengths	Template sequence	1968	75	263	188	1291	151
	Population sequence	1935–1936				1258–1259	
Polymorphic sites	Invariant site	1923	75	255	186	1251	150
	Polymorphic sites	12	0	0	2	7	0
	Parsimony informative sites	12			1	7	0
	InDel	0			1	0	
***Fel d1 CH2***							
Nucleotide sequence lengths	Template sequence	2230	68	513	182	1252	215
	Population sequence	2226–2233				1248–1255	215–221
Polymorphic sites	Invariant site	2172	68	504	173	1222	202
	Polymorphic sites	51	0	9	9	23	13
	Parsimony informative sites	51	0	9	9	13	13
	InDel	15	0	0	0	8	7

Pi: Nucleotide polymorphism; K: average nucleotide difference; and InDel: insertion/deletion. Total sequence, introns, and exons are presented as the number of base pairs.

**Table 8:** Polymorphism analysis of *Fel d1* gene coding sequence.

Gene Name	Pi	K	Polymorphic Sites	InDel
*CH1*	0.00185	0.495	2	0
*CH2*	0.02193	7.1	18	7

Pi: Nucleotide polymorphism; K: average nucleotide difference; and InDel: insertion/deletion.

## Data Availability

Data are contained within the article.
